# Flexibility in joint coordination remains unaffected by force and balance demands in young and old adults during simple sit-to-stand tasks

**DOI:** 10.1007/s00421-018-4035-4

**Published:** 2018-11-24

**Authors:** Christian Greve, Tibor Hortobágyi, Raoul M. Bongers

**Affiliations:** 1 Center for Human Movement Science, University of Groningen, University Medical Center Groningen, Hanzeplein 1, HPC CB41, Postbus 30.001, 9700 RB Groningen, The Netherlands; 2 Department of Rehabilitation Medicine, University of Groningen, University Medical Center Groningen, Groningen, The Netherlands

**Keywords:** Motor control, Ageing, Motor flexibility, Coordination, Uncontrolled manifold, Sit-to-stand

## Abstract

**Purpose:**

We examined the possibility that old adults use flexibility in joint coordination as a compensatory mechanism for the age-related decline in muscle strength when performing the sit-to-stand (STS) task repeatedly under high force and balance demands.

**Method:**

Young (*n* = 14, 22.4 ± 2.1) and old (*n* = 12, 70 ± 3.2) healthy adults performed repeated STSs under high and low force and balance demands. The balance demand was manipulated by reducing the base of support and the force demand by increasing body weight with a weight vest. Uncontrolled manifold analysis was used to quantify age differences in motor flexibility.

**Results:**

While there were age-typical differences in kinematic STS strategies, flexibility in joint coordination was independent of age and task difficulty during repeated STSs.

**Discussion:**

That simple manipulations of force and balance demands did not affect flexibility in joint coordination in old and young adults suggests that motor flexibility acts as a compensatory mechanism only at the limits of available muscle strength and balance abilities during STS movements. Intervention studies should identify how changes in specific neuromuscular functions affect flexibility in joint coordination during activities of daily living such as STS.

## Introduction

Standing up from a chair or bed is a frequent activity of daily living and requires muscle strength, power, and balance (Dall and Kerr 2010; Hughes et al. 1994, 1996; Riley et al. 1991). In compensation for the age-related decline in neuromuscular function, old adults compared with young adults adjust their movement patterns and execute the sit-to-stand (STS) tasks more slowly, with a greater trunk flexion, and with lower peak vertical ground reaction forces at lift-off (Alexander et al. 2001; Gross et al. 1998; Hughes et al. 1994, 1996; Yamada and Demura 2010). Beyond such biomechanical adjustments, old adults can also rely on inter-joint coordination during the STS task and increase co-variation among lower and upper extremity joints, the trunk and the neck to seek stability (Greve et al. 2013). Here, we explore the idea that old adults use multi-joint co-variation in compensation for muscle weakness when the STS task becomes difficult and for instability caused by reductions in the base of support.

During STS movements, the lower- and upper-extremity joints, the trunk, and the neck are coordinated to bring the whole body center of mass (COM) from a low to a high position. Because there are more degrees of freedom (> 8 independent joint motions) than three dimensional constraints to the COM position, the STS task can be performed with different joint configurations (Gelfand and Latash 1998; Greve et al. 2013; Latash 2012; Latash et al. 2007; Scholz and Schöner 1999). During task execution old and young adults make use of this abundance in the movement repertoire and adapt the STS movement to external (e.g., chair height), and internal (e.g., muscle weakness, instability) constraints (Newell 1986; Hu and Newell 2011) through small and coordinated adjustments among all available joint motions. This flexibility in joint coordination underlies multi-joint co-variation and guarantees safe COM positions during daily-life STS performance where the actual constraints to movement are poorly predictable, and might even change. This concept of performance stability through flexibility in joint coordination has been previously described and tested in STS and other motor tasks (Domkin et al. 2002; Eckardt and Rosenblatt 2018; Golenia et al. 2018; Greve et al. 2013; Latash 2012; Latash et al. 2007; Olafsdottir et al. 2007; Scholz and Schöner 1999; Wu et al. 2013).

When raising the COM from a low to a high point, the lower-extremity extensor muscles generate force at an unfavorable point on the length-tension curve, which, combined with muscle weakness, makes the STS task a near maximal effort in old adults (Alexander et al. 1991, 2001; Gross et al. 1998; Hortobágyi et al. 2003). In compensation for this high muscular effort, old adults increase trunk flexion and bring the whole body COM closer to the knee at lift-off (Harris and Wolpert 1998). This adaptation in STS strategy minimizes the range of possible COM positions, which old adults can use at lift-off. Having a smaller range of possible COM positions at lift-off increases the risk of task failure or a fall in case of changes in internal or external constraints such as a perturbation. In addition, muscle weakness can also challenge COM stability because generating near-maximal muscle forces increases variability in force output (Harris and Wolpert 1998; Jones et al. 2002; Schmidt et al. 1979; Slifkin and Newell 1999). After peak trunk flexion a well-timed and rapid knee extensor muscle burst initiates the extension movement of the lower extremities and trunk (Lindemann et al. 2007; Riley et al. 1991; Scarborough et al. 2007). High variability in knee extensor force output de-stabilizes the transition from horizontal to vertical COM motion at lift-off. We examined the idea that old adults maintain COM stability despite strength deficits by increasing co-variation among the involved joint motions when force and balance demands are high during STS performance.

In line with this idea we found in a previous study that old adults, as compared to young adults, increase co-variation among the lower- and upper-extremity joints, the trunk and the neck to improve COM stability at lift-off when repeatedly performing the same STS task (Greve et al. 2013). We proposed that this age-related increase in motor flexibility reflected a compensatory mechanism for strength and balance deficits. However, we did not manipulate force and balance requirements, making it impossible to discern if strength and balance deficits interacted with motor flexibility. Thus, the purpose of the present study was to determine the effects of age and task difficulty on flexibility in joint coordination. We manipulated task difficulty by having participants perform the STS task with extra weight and over a reduced base of support under the feet.

Based on the age-related decline in muscle strength and balance abilities, and the retained ability in old age to exploit motor flexibility (Greve et al. 2013), we expected that old adults increase multi-joint co-variation to employ a larger range of equivalent coordination patterns when performing repeated STS tasks under high (a) force, (b) balance, and (c) force and balance demands. We hypothesized that when balance and force demands are high, old but not young adults increase the range of those joint configurations stabilizing the COM position between trials.

## Method

### Participants and design

Healthy old and young adults free of self-reported neurological or musculoskeletal disorders in the upper and lower extremities participated in the study (Table 1). Participants had normal vision with or without a correction.

**Table 1:** Participant characteristics

	Young (*n* = 14, male = 5)		Old (*n* = 13, male = 5)	
	Mean	SD	Mean	SD
Age (years)	22.4	2.1	70.0	3.2
Height (m)	1.9	0.3	1.7	0.1
Weight (kg)	70.2	13.2	74.8	13.3
VAS (0–100)	26.4	10.6	38.5	30.5
Knee extensor (N/kg)**				
Pre	5.3	0.3	3.0	0.3
Post	5.4	0.3	3.2	0.3
Knee flexor (N/kg)**				
Pre	3.0	0.2	2.0	0.2
Post	3.1	0.2	2.0	0.2
SPPB				
Total**	12.0	0.0	11.0	1.0
Balance	4.0	0.0	3.8	0.4
STS**	4.0	0.0	3.2	0.8
Gait	4.0	0.0	4.0	0.0

**p < 0.001 for main effects of age

The difficulty of the STS task was manipulated by increasing the balance and force demands, resulting in four experimental conditions: (1) low force and low balance demands, (2) high force and low balance demands, (3) low force and high balance demands, and (4) high force and high balance demands. Before the start of each new STS condition, participants performed three-to-five familiarization trials. After familiarization participants performed 25 STS trials for each experimental condition resulting in a total of 100 chair-rises per participant. The order of the conditions was randomized between participants.

### Experimental set-up

**Fig. 1:**
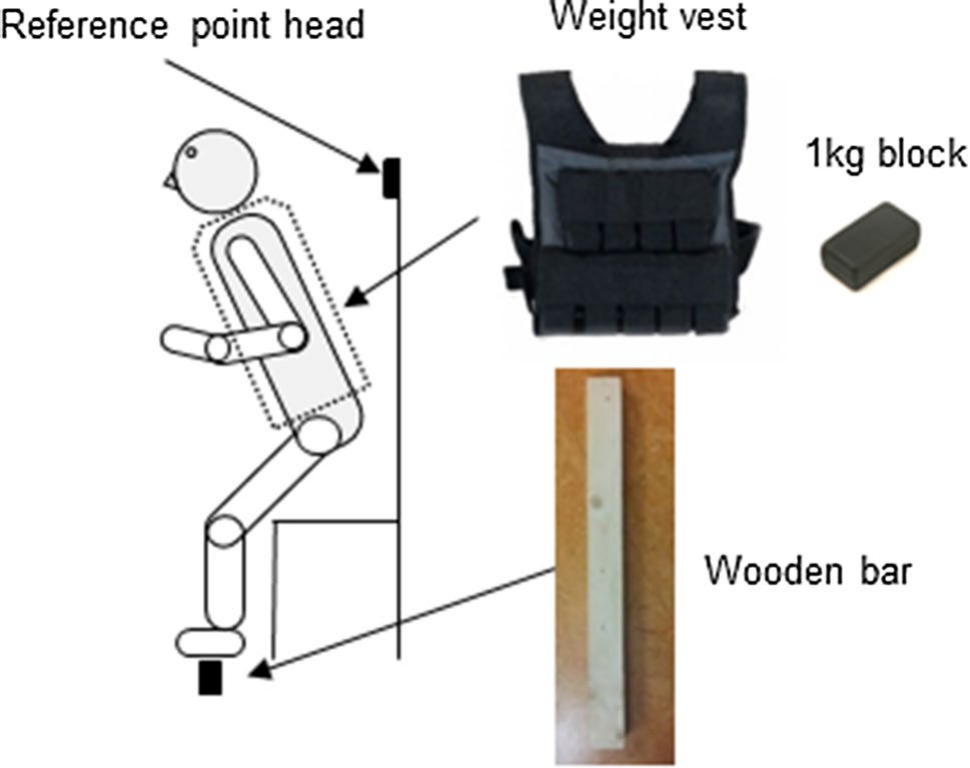
Experimental set-up

Participants stood up from an armless chair set at 110% of lower leg length using the fibula head as reference (Fig. 1). Before the start of the experiment, participants were instructed to sit upright and place their hands on the thighs and the feet on the force plate in front of the chair. Participants were free to choose their foot placement for each condition but had to maintain the same feet position between consecutive repetitions of each STS condition. The initial foot position was marked by tape on the force plate just next to the fifth metatarsal and behind the calcaneus to assure the same feet position between consecutive trials. Furthermore a wooden stick was placed at the height of each participants’ occipital bone and used as a reference point to assure the same upright position between consecutive trials. Balance demands were manipulated by varying the width of the participants’ support surface with a wooden bar. The width of the support surface was scaled to each participant’s shoe size (0.27 × European shoe size). To manipulate the strength demand participants wore a weight vest where 1 kg heavy blocks could be added to increase the total weight. The added weight was scaled to the averaged maximum voluntary contraction (MVC) values of the right knee extensor muscles and distributed symmetrically on the back and front of the weight vest. During the low-strength demand condition (0%) participants did not wear the weight vest and during the high-strength demand condition (30%) participants wore the weight vest. The added weight was distributed evenly on the ventral and dorsal side of the trunk. The scaling factor of 0.27 for the support surface width and the added weight of 30% MVC for the force demand were based on a pilot experiment in four healthy young participants.

Synchronized measurements were done using a Kistler force plate (Bertec, Columbus, OH, USA), an Optotrak motion capture system (Northern Digital Inc., Waterloo, Ontario, Canada), and an electromyographic (EMG) system (Trigno, Delsys Inc, Natick, MA, USA). The force plate recorded 3D ground reaction forces and moment of forces at 1 kHz. The Optotrak motion capture system consisted of two units recording positions of eight markers at 100 Hz. The markers were attached to the right side of the participants body on the base of the fifth metatarsal, lateral malleolus, lateral femoral epicondyle, greater femoral trochanter, inferior-to-lateral aspect of acromion process, lateral humeral epicondyle just superior to radiohumeral junction, styloid process of radius and immediately anterior to external auditory meatus. We recorded EMG signals of the rectus femoris, vastus medialis, semimembranosus, gastrocnemius, soleus and tibialis anterior muscles using 37 × 27 × 15 mm, < 15 g, wireless, pre-amplified (909x) parallel-bar sensors, affixed to the skin with a four-slot adhesive skin interface (Trigno, Delsys Inc, Natick, MA, USA). The EMG signals were sampled at 4 kHz. Before electrode placement the subject’s skin was shaved, scrubbed with fine sandpaper and cleaned with alcohol to minimize noise in the EMG signal. The electrodes recorded with a bandwidth of 20–450 Hz, channel noise < 0.75 µV, and common-mode rejection ratio > 80 dB. Signals were acquired on-line and stored by software installed on a personal computer for off-line analysis (Signal, Cambridge Electronics Design, Cambridge, UK).

### Experimental procedures

Before the main experiment, each participant performed the short physical performance battery to assess balance and mobility (Guralnik et al. 1994). Next, three trials of 4-s-long MVCs for the knee flexor and extensor muscles were performed against a load cell of a hand-held dynamometer (ErgoFet, Hoggan Health Industries, West Jordan, USA). During the MVC measurements participants sat upright on a table with the knees at 90°. The load cell was hand-held by a trained technician, and placed at the front of the distal tibia to resist the extension movement, and at the back of the distal tibia to resist the knee flexion movement. Participants rested for 30–60 s between consecutive MVCs. The MVC measurements were repeated after the experiment to establish whether participants fatigued during the experiment. The weight added during the force demand condition was defined as 30% of the knee extensor MVC value averaged across the three trials.

During the experiment, participants were instructed to rise from a chair 25 times within a condition at a self-selected pace. Participants were free to reposition their arms but were not allowed to push with their hands on the thighs. Before each repetition the start position was checked and corrected as needed. Participants started the STS series in response to an auditory cue. The investigator emphasized that the STS task was not a reaction time task and that participants could initiate the STS movement after the first auditory cue. After initiation of the STS movement participants remained in a standing position for 2 s until a second auditory cue occurred, which prompted participants to sit down and continue the STS task. Participants were allowed to pause, rest, and drink water between consecutive conditions. Before the start of the experiment, the investigator asked the participants if they understood the task correctly. There were 2 min of rest between conditions. The entire experiment lasted 60–90 min per participant, including preparation.

### Data analysis

STS trials during which one or more markers were invisible 100 ms before or after lift-off were excluded from analysis. Lift-off was determined as the instant when trunk flexion changed to extension (Zijlstra et al. 2012). On average 21 ± 3.1 trials per participant were used in the final analyses. Coordinate data of each marker and force plate data were filtered using a bi-directional fourth-order low-pass Butterworth filter with a cutoff frequency of 6 Hz. Marker coordinates were processed to calculate segment angles with the horizontal in the sagittal plane of the foot, ankle, knee, trunk, head, shoulder, elbow and wrist. Sagittal plane knee joint moments were computed using linear and angular Newtonian equations (Enoka 2008; Winter 2009). Peak knee joint moments were used to estimate the required knee extensor muscle force at lift-off and allow comparability of force demands between studies and conditions. EMG data were rectified, high-pass filtered at 2 Hz and then low-pass filtered at 10 Hz using a bi-directional 4th order Butterworth filter.

As an additional clinical measure, we computed the amount of co-activation of the agonist antagonist muscles of the upper and lower leg muscles according to Winter (2009):

$$\% {\text{Co-contraction}}=2 \times \frac{{{\text{common area }}({\text{A and B}})}}{{{\text{area A}}+{\text{area B}}}} \times 100$$

where A is the agonist muscle and B the antagonist muscle. The percentage of co-contraction was calculated over a time window of 100 ms. This time window started 50 ms seconds before peak trunk flexion and ended 50 ms after peak trunk flexion. The common area (A and B) reflects the common area under the EMG curve where both muscles are active simultaneously. Area A and area B reflects the area under the EMG curve of the antagonist and the agonist muscle individually.

The sagittal plane CoM position in anterior–posterior and vertical direction was calculated based on participants’ body-segment lengths and the estimated locations and proportions of segmental masses (Winter 2009). A detailed description of the formula is given in Scholz and Schöner (1999) and Greve et al. (2013) (Winter 2009). Seven segmental angles with the horizontal [foot, shank, thigh, trunk, upper arm (ua) and lower arm (la)] were used to compute the CoM position in the sagittal plane. Grand means of segmental length based on all trials to be representative of a constant segmental length were used. CoM velocity was computed based on the time derivative. CoM variability was defined as the across-trial standard deviation of the 2D vector length of the CoM position at lift-off.

Customized MATLAB scripts were used for the analysis (MATLAB R2012). Duration of each sit-to-stand trial was determined by the initiation of forward trunk movement and end of trunk motion, defined by a threshold of angular change of 0.009 radians within 0.05 s (Zijlstra et al. 2012). Accuracy of the algorithm in event detection was visually controlled for each sit-to-stand trial.

### Age differences in motor flexibility

To establish age differences in motor flexibility, we performed uncontrolled manifold (UCM) analysis based on the co-variance matrix C. UCM analysis allowed us to decompose trial-to-trial variability in elemental variables into multi-joint co-variation stabilizing the COM at its’ desired position between trials (GEV) and multi-joint co-variation leading to small changes of the COM position between trials (NGEV). A detailed description of UCM analysis can be found elsewhere (Neilson and Neilson 2010; Tuitert et al. 2017; van der Steen and Bongers 2011; Verrel 2011; Scholz and Schöner 1999; Greve et al. 2013). Segment angles of the foot, lower and upper leg, trunk, lower and upper arm, hand and head were used as elemental variables and the sagittal plane COM position at lift-off as performance variable. We chose the whole body COM position and not the vertical ground reaction force (GRF) vector (Greve et al. 2013) as performance variable because during the manipulation of the support surface during the balance demand conditions, the GRF vector could not be reliably measured. The same geometric model as previously published was used to compute the Jacobian (J) (Scholz and Schöner 1999; Greve et al. 2013).

### Statistical analysis

We established how young and old adults make use of the available motor flexibility during repeated STS performance with a repeated measures ANOVA on UCM measures with variability component (GEV and NGEV), balance demand (normal and small base of support) and force demand (0% and 13% MVC) as within subjects factor and age (young and old) as between subjects factor. UCM measures were log-transformed before statistical analysis.

Age differences in overall perceived difficulty of the STS experiment, muscle strength and fatigue were established with repeated measures ANOVAs on VAS scores (visual analogue scale from 0 = not difficult at all to 10 = very difficult) and strength profiles with age as between subjects factor and measurement (pre- and post-experiment) as within-subject factor for the fatigue analysis.

How young and old adults adapted their kinematic behavior and muscular co-activation profiles when force and balance demands increased was analyzed with five repeated measures ANOVA on duration, peak trunk flexion, peak COM velocity, across-trial standard deviation of the COM, peak vertical GRFs normalized by body weight and the percentage of agonist antagonist muscle co-activation. The across-trial standard deviation of the COM position was based on the two dimensional vector length of the anterior–posterior and vertical CoM position. Age was selected as between-subjects factor and force and balance demand as the within-subjects factor. Analysis of the peak vertical GRF data was only performed during the normal support surface conditions. All statistical analyses were performed with SPSS 24.0.

## Results

### Participant characteristics

14 young (22.4 ± 2.1 years) and 12 old (70 ± 3.2 years) participants were included in the final analysis. Table 1 shows participant characteristics. Old compared with young adults had less peak knee extensor and knee flexor muscle strength (knee extension *p* < 0.001, *F*_1,25_ = 36.7; knee flexion *p* = 0.001, *F*_1,25_ = 14.9; Table 1) but represented good balance and overall physical performance capacities. Old and young adults similarly perceived the difficulty of the experiment as low-to-moderate.

### UCM analysis

The amount of stabilizing (GEV) as compared to de-stabilizing (NGEV) joint coordination patterns was significantly larger [GEV(log(rads^2^)) − 5.3 ± 0.12; NGEV (log(rads^2^)) − 6.7 ± 0.12; *p* < 0.001, *F*_1,24_ = 194.6] but not different between age groups (variability × age *p* = 0.798, *F*_1,24_ = 0.07). The total amount of variability increased when standing up from smaller support surface as reflected by a main effect for balance demand independent of age (low balance demand − 6.3 ± 0.1; high balance demand − 5.8 ± 0.14; *p* = 0.001, *F*_1,24_ = 16.1). Note that the higher total amount of variability was evenly distributed into GEV and NGEV (variability × balance demand *p* = 0.466, *F*_1,24_ = 0.55). Based on our previously published paper, we expected that GEV would increase more than NGEV in the old but not young adults when force demands were higher (Greve et al. 2013). However, the old and young adults employed similar GEV and NGEV during the low and high physical demand conditions (physical demand × variability *p* = 0.67, *F*_1,24_ = 0.19).

### Age differences in STS strategies

Similar to previous studies the old as compared to young adults performed the STS movements slower (young 1.7 ± 0.05 s; old 2.0 ± 0.05 s; *p* < 0.001, *F*_1,24_ = 18.1) and employed larger trunk flexion at lift-off (young 56.8° ± 2.3°; old 52.6° ± 2.5°); however, this age difference was not significant (*p* = 0.22, *F*_1,24_ = 1.6; Tables 2, 3). When weight was added by the weight vest the old and young adults adapted to the higher force requirements by slowing down STS performance (*p* < 0.01, *F*_1,24_ = 9.5; Table 2). Peak trunk flexion angles did not increase when standing up under higher force requirements (*p* = 0.29, *F*_1,24_ = 1.2; Table 3). When standing up from smaller support surface sizes both groups slowed down (*p* < 0.001; *F*_1,24_ = 26.3; Table 2) and flexed the trunk more at lift-off (*p* < 0.001; *F*_1,24_ = 18.1; Table 3). Trial-to-trial variability of the 2D COM position at lift-off was similar between age groups (old 0.11 ± 0.01 m, young 0.11 ± 0.01 m; *p* = 0.93; *F*_1,24_ = 0.01) and increased with increases in balance but not force demands (balance low 0.09 ± 0.01 m; balance high 0.13 ± 0.01 m; *p* < 0.01; *F*_1,24_ = 8.7).

**Table 2:** Sit-to-stand duration (s)

	Young		Old	
	Mean	STD	Mean	STD
Age**	1.69	0.05	1.98	0.05
Balance**				
Low	1.64	0.05	1.93	0.05
High	1.75	0.05	2.04	0.06
Force*				
Low	1.66	0.05	1.97	0.05
High	1.73	0.05	2.01	0.05

*p < 0.01 or **p < 0.001 for main effects of age, balance, and force

**Table 3:** Peak trunk flexion angles at lift-off (angle with horizontal)

	Young		Old	
	Mean	STD	Mean	STD
Balance**				
Low	58.5	2.1	55.0	2.2
High	54.1	2.7	50.3	2.9
Force				
Low	58.5	2.1	55.0	2.2
High	59.7	2.2	55.4	2.4

**p < 0.001 for main effects of balance

Analysis of kinetic STS behavior did not reveal any age differences. Table 4 shows that peak GRF and knee joint moments normalized by body weight did not differ between age group. Due to technical problems in the center of pressure detection, peak GRF and knee joint moments were obtained in 10 instead of 12 old participants.

**Table 4:** Kinetic motor behavior (N m/kg)

	Force (%)	Young		Old	
		Mean	STD	Mean	STD
GRF vertical (N/kg)^a^	0	11.5	0.19	11.8	0.25
	13	13.1	0.21	13.2	0.27
Knee moment (Nm/kg)^a^	0	0.33	0.02	0.38	0.02
	13	0.32	0.02	0.38	0.02

^a^Normalized by body weight (kg), *p* = 0.03

### Agonist–antagonist muscle co-activation

Old and young participants performed the STS task with similar agonist–antagonist muscle co-activation of the upper and lower leg (young 60.5 ± 2.9%, old 64.9 ± 3.1%; *F*_24,1_ = 1.06, *p* = 0.31). Muscular co-activation did not interact with the balance and force requirements of the STS task.

## Discussion

We aimed to establish whether healthy old adults make use of flexibility in joint coordination to compensate for the age-related decline in knee extensor muscle strength and guarantee safe STS performance when force and balance demands are high. Contrary to the hypothesis, old and young adults similarly employed multi-joint co-variation to stabilize the COM at lift-off when repeatedly standing up from a chair under varying force and balance demands. Interestingly, old adults stood up from a chair slower than young adults and although we observed main effects of the force and balance demands, there were no differences in kinematic adaptations to force and balance demands between the two age groups. Because maximal voluntary force did not decrease after repeated STS, fatigue did not represent an additional physical demand.

When humans perform voluntary movements such as STS, reaching, or standing balance, the abundant joint motions provide the neuromuscular system with a large range of movement possibilities (Gelfand and Latash 1998; Latash 2012; Latash et al. 2007; Scholz and Schöner 1999). During task execution, coordination patterns emerge based on external and internal constraints allowing old and young adults to adapt to changes in task requirements or even small perturbations (Hu and Newell 2011; Newell 1986; Newell and Verhoeven 2017). This flexibility in joint coordination underlies multi-joint co-variation and therefore guarantees COM stability at lift-off independent of changes in the actual joint configurations.

When healthy old adults rise from a low chair, they use 80–100% of the available knee extensor torque compared to 40–60% in healthy young adults (Hughes et al. 1996; Lindemann et al. 2007). Operating at the limits of intrinsic force capacities requires more accurate COM control closer to the knee joint in the old as compared to young adults at lift-off (Alexander et al. 2001; Hughes et al. 1996; Winter 2009). In a previous STS experiment we showed that old adults who have low knee extensor muscle strength, increased co-variation among the available joint motions. A stronger coupling among the available degrees of freedom allows the neuromuscular system to use a larger range of the available movement possibilities while guaranteeing STS stability (GEV) (Greve et al. 2013). The current experiment elaborated on this finding and examined the idea whether the observed increase in motor flexibility compensated for task difficulty in the STS task, created by deficits in intrinsic strength constraints (added weight) or balance abilities (reductions in base of support).

Our old as compared to young participants had similar balance and overall physical performance capacities but old adults’ quadriceps muscle was 38% weaker (*p* < 0.01; Table 1). Due to this deficit in knee extensor muscle strength our old adults performed slower (Table 2), adaptation in STS strategy similar to previous studies (Alexander et al. 2001; Gross et al. 1998; Hughes et al. 1994, 1996; Yamada and Demura 2009). To establish whether old adults increased multi-joint co-variation in compensation for strength or balance deficits, we manipulated force and balance demands of the STS task by increasing body weight and reducing base of support. The rationale was that higher force and balance constraints would interact with reductions in voluntary muscle strength requiring old adults to stabilize the whole body COM position within a smaller range at lift-off. Based on the principle of motor abundance we expected that old adults would increase co-variation among the available joint motions to guarantee accurate and safe positioning of the whole body COM position at lift-off. This stronger coupling would allow old adults to perform STS movements safely and adapt to changes in task requirements despite deficits in muscle strength or balance. Hence, increasing flexibility in the abundant joint motions was suggested to be a mechanism allowing healthy old adults to successfully perform STS movements in daily-life environments (Gelfand and Latash 1998; Latash 2012, 2016; Latash et al. 2007, 2010).

However, task difficulty did not affect flexibility in joint coordination in either age group while execution speed of STS decreased with increases in force and balance demands and trunk flexion increased at lift-off with increases in balance demands (Tables 2, 3). Reducing movement speed and bringing the COM closer to the knee at lift-off are effective strategies to improve COM stability (Hughes et al. 1994, 1996; Lindemann et al. 2007). Hence, these adaptations in movement kinematics might have been sufficient to guarantee safe STS performance in young and old adults under the tested force and balance manipulations. Considering the current data and our previous results on age differences in motor flexibility during STS movements, the possibility exists that flexibility in joint coordination might only be employed when old adults operate at the limits of the available muscle strength or balance abilities (Greve et al. 2013).

The difference between the present and past study was that in this study participants stood up from higher chair heights (110 vs. 100% of lower leg length) and used lower peak knee extension moments at lift-off (110%: young 0.32 ± 0.02; old 0.38 ± 0.02 vs. 100%: young 2.24 ± 0.29; old 2.02 ± 0.25 N/kg). Especially low chair heights impose high force constraints because the origin and insertion of the rectus femoris muscle are closer to each other leading to an unfavorable force–length relationship (Gerritsen et al. 2016; Winter and Challis 2010). In addition to the lower force requirements, our old and young adults had good balance and overall physical performance capacities (Table 1). Physical performance capacities and balance abilities were not measured in the previous experiment. Therefore, the possibility exists that task difficulty was not high enough to cause an interaction between age and intrinsic force and balance abilities. Only when old and young adults operate more at the limits of their intrinsic strength and maybe balance capacities flexibility in joint coordination might be used to stabilize STS performance.

In sum, age and task difficulty of the STS task did not affect motor flexibility. It is well-established that in addition to deficits in muscle strength and power (Faulkner et al. 2007; Thompson 2009) healthy ageing affects various aspects of the neuromuscular system. For example, old as compared to young adults have an impaired ability to integrate proprioceptive feedback (Goble et al. 2009) and to coordinate agonist–antagonist muscle pairs (Hortobágyi and Devita 2006). Furthermore the central nervous system suffers from a decline in number and size of afferent fibers (Romanovsky et al. 2015), less cortical and spinal neurons (Dinse 2006; Eisen et al. 1996; Erim et al. 1999), a reduction in motor cortical inhibition (Hortobágyi et al. 2006; Papegaaij et al. 2014; Peinemann et al. 2001) and cognitive dysfunctions involved in postural control (Morris et al. 2016; Seidler et al. 2011; Woollacott and Shumway-Cook 2002). Based on these well-established findings on age differences in neuromuscular functions previous studies assumed that flexibility in joint coordination might also decline with ageing. However, our current findings and previous studies on age differences in motor flexibility during STS and other tasks provide evidence that there is not a general decline in motor flexibility with ageing (Decker et al. 2012; Eckardt and Rosenblatt 2018; Freitas and Duarte 2012; Greve et al. 2013, 2015, Hsu et al. 2013, 2014; Kapur et al. 2010; Krishnan et al. 2013; Krüger et al. 2013; Olafsdottir et al. 2007; Shim et al. 2004; Singh et al. 2013; Skm et al. 2012; Verrel et al. 2012; Wu et al. 2013; Xu et al. 2013).

Overall, previous studies investigating age differences in motor flexibility during reaching, gait, multi-finger force coordination and postural tasks report inconclusive findings (Decker et al. 2012; Eckardt and Rosenblatt 2018; Freitas and Duarte 2012; Greve et al. 2013, 2015, Hsu et al. 2013, 2014; Kapur et al. 2010; Krishnan et al. 2013; Krüger et al. 2013; Mattos et al. 2011; Olafsdottir et al. 2007; Shim et al. 2004; Singh et al. 2013; Skm et al. 2012; Verrel et al. 2012; Wu et al. 2013; Xu et al. 2013). During postural tasks and gait, larger motor flexibility was reported in old as compared to young adults when standing up from low chair heights (Greve et al. 2013), whereas less motor flexibility was observed in old adults during a balance perturbation and narrow base standing task (Hsu et al. 2013, 2014). No age differences in motor flexibility occurred during quiet standing, normal gait, gait on uneven surfaces, and a dual cognitive and motor task on a treadmill (Decker et al. 2012; Freitas and Duarte 2012, Hsu et al. 2014, Hsu and Scholz 2012; Krishnan et al. 2013). During simple reaching movements Verrel et al. (2012) reported poorer whereas Krüger et al. (2013) reported greater and Xu et al. (2013) and Greve et al. (2015) similar motor flexibility in old as compared to young adults (Greve et al. 2015; Krüger et al. 2013; Verrel et al. 2012; Xu et al. 2013). Similar inconclusive findings were reported by studies investigating flexibility in multi-finger force coordination tasks (Kapur et al. 2010; Olafsdottir et al. 2007; Shim et al. 2004; Singh et al. 2013; Varadhan et al. 2012; Wu et al. 2013). Some authors reported less flexibility in force coordination (Kapur et al. 2010; Olafsdottir et al. 2007; Shim et al. 2004), whereas others reported no age differences (Singh et al. 2013; Skm et al. 2012) and others even larger motor flexibility when the agonist finger muscles were fatigued, and after practicing a challenging coordination task (Singh et al. 2013; Wu et al. 2013). Summarizing, the current data on age differences in motor flexibility do not allow us to conclude whether and if so how healthy ageing affects flexibility in joint coordination and multi-finger force control. We propose that complex interactions between age-related deficits in neuromuscular functions with the task requirements and constraints imposed by the experimental environment affect young and old adults’ coordination strategies leading to significant differences during even similar movements.

The finding that old as compared to young adults use more of the available motor flexibility when force requirements are high during STS (Greve et al. 2013), under conditions of fatigue and with practice of challenging multi-finger force coordination tasks (Singh et al. 2013; Wu et al. 2013) combined with our current data suggest that motor flexibility might serve in compensation for age-related deficits in neuromuscular functions only when operating at the maximum of the available neuromuscular capacities. To further augment our understanding of how the processes of healthy ageing affect motor flexibility, future studies should perform longitudinal analyses in well-controlled experiments. We should aim to establish how changes in specific neuromuscular functions (e.g., muscle strength) interact with given environmental and task constraints of motor tasks frequently performed in daily life. Finally, this knowledge can be used to effectively design prevention and rehabilitation programs in old adults with and without pathology.

We also note the possibility that standardizing the start position of the joints prior to movement initiation might have confined motor flexibility. During our experiment the participants were instructed to repeatedly perform the STS movement from the same start position without repositioning their feet between trials. During unconstrained STS movements old participants with strength deficits might have chosen to adapt their feet position before movement initiation to adapt to higher force and balance demands. Therefore, fixing the start position might have constrained the use of the available flexibility in joint coordination.

## Limitations

One limitation is that the analysis focused on the sagittal plane of the STS task and we cannot tell how healthy ageing affects flexibility in joint coordination in the frontal plane during repeated STS performance. Furthermore, our experimental constraints might have restrained the extent to which participants could explore the available motor flexibility possibly leaving effects of task demand on flexibility in joint coordination undetected.

## Conclusion

Flexibility in joint coordination remains unaffected by age and increases in force and balance demands during repeated STS performance. Only when old and possibly young adults operate at the limits of their available muscle strength and balance flexibility in joint coordination might act as a compensatory mechanism to stabilize STS performance. Current data and previous results imply that old age does not lead to a universal decline in motor flexibility.

## Abbreviations

ADL: Activities of daily living
ANOVA: Analysis of variance
BOS: Base of support
COM: Center of mass
DOF: Degrees of freedom
EMG: Electromyography
GEV: Goal-equivalent variability
GRF: Ground reaction force
MVC: Maximal voluntary contraction
NGEV: Non-goal-equivalent variability
STD: Standard deviation
STS: Sit-to-stand
UCM: Uncontrolled manifold
VAS: Visual analogue scale
